# Unraveling the Molecular Mechanisms Involved in HCV-Induced Carcinogenesis

**DOI:** 10.3390/v14122762

**Published:** 2022-12-11

**Authors:** Tania Guadalupe Heredia-Torres, Ana Rosa Rincón-Sánchez, Sonia Amelia Lozano-Sepúlveda, Kame Galan-Huerta, Daniel Arellanos-Soto, Marisela García-Hernández, Aurora de Jesús Garza-Juarez, Ana María Rivas-Estilla

**Affiliations:** 1 Department of Biochemistry and Molecular Medicine, CIIViM, School of Medicine, Universidad Autónoma de Nuevo León (UANL), Monterrey 64460, Mexico; 2 IBMMTG, Departamento de Biología Molecular y Genómica, Centro Universitario de Ciencias de la Salud, Universidad de Guadalajara, Guadalajara 44100, Mexico

**Keywords:** hepatitis C virus, carcinogenesis, hepatocellular carcinoma, inflammation, proliferation, epithelial-mesenchymal transition

## Abstract

Cancer induced by a viral infection is among the leading causes of cancer. Hepatitis C Virus (HCV) is a hepatotropic oncogenic positive-sense RNA virus that leads to chronic infection, exposing the liver to a continuous process of damage and regeneration and promoting hepatocarcinogenesis. The virus promotes the development of carcinogenesis through indirect and direct molecular mechanisms such as chronic inflammation, oxidative stress, steatosis, genetic alterations, epithelial-mesenchymal transition, proliferation, and apoptosis, among others. Recently, direct-acting antivirals (DAAs) showed sustained virologic response in 95% of cases. Nevertheless, patients treated with DAAs have reported an unexpected increase in the early incidence of Hepatocellular carcinoma (HCC). Studies suggest that HCV induces epigenetic regulation through non-coding RNAs, DNA methylation, and chromatin remodeling, which modify gene expressions and induce genomic instability related to HCC development that persists with the infection’s clearance. The need for a better understanding of the molecular mechanisms associated with the development of carcinogenesis is evident. The aim of this review was to unravel the molecular pathways involved in the development of carcinogenesis before, during, and after the viral infection’s resolution, and how these pathways were regulated by the virus, to find control points that can be used as potential therapeutic targets.

## 1. Introduction

Cancer is one of the leading causes of death worldwide. It is estimated that around 1,400,000 annual cancer cases are caused by viral infections. Liver cancer is the second most common cause of cancer mortality and the seventh most frequently occurring cancer in the world [1]. The principal type of liver cancer is hepatocellular carcinoma (HCC). One of the most important global risk factors for HCC is a Hepatitis C virus (HCV) infection, and there are globally estimated 71.1 million people infected [2,3]. Vaccination programs are the principal prevention strategy for viral infections; unfortunately, there is no vaccine to prevent an HCV infection. Despite the development of direct-acting antivirals (DAAs) with a sustained virological response (SVR) against HCV, it has been reported that patients treated with DAAs presented an unexpected increase in the incidence of HCC. The aim of this review was to unravel molecular mechanisms involved in the development of carcinogenesis before, during, and after the viral infection resolution. The understanding and crosstalk of the diverse molecular mechanisms, such as proliferation, oxidative stress, resistance to apoptosis, and epithelial-mesenchymal transition, among others, induced by viral infections are essential to offer improvements both in antiviral treatment and in the prevention of the development of carcinogenesis.

## 2. Viral-Infections-Induced Human Cancers

To date, there are considered to be seven human oncoviruses: Epstein–Barr virus (EBV), hepatitis B virus (HBV), Hepatitis C virus, human papillomaviruses (HPV), human T-cell lymphotropic virus (HTLV-1), Kaposi’s sarcoma-associated herpesvirus (KSHV), and Merkel cell polyomavirus (MCPyV) [4]. The characteristics and tropisms of oncoviruses vary widely, from naked MCPyV, with a circular DNA genome that is attributable to 80% of Merkel cell carcinomas, to HCV, an enveloped virus with positive-sense RNA that is attributable to 3% of Non-Hodgkin lymphomas [1]. One of the main advantages of studying each oncogenic virus and its mechanisms is to avoid the development of carcinogenesis by preventing viral infection. However, each oncovirus has important oncogenic factors and mechanisms that are primarily attributable to the development of carcinogenesis (Table 1). Through the study and understanding of these factors and mechanisms, effective vaccines and treatments have been developed to prevent or treat infections and to reduce the incidence of the associated cancers. However, HCV and the risk of HCC developing after an infection’s clearance demonstrate that identifying the virus as carcinogenic in origin is not enough to establish alternatives to cancer prevention. Therefore, it is extremely important to study how a viral infection can regulate processes from altered proliferation, apoptosis resistance, cell cycle arrest, and oxidative stress to the processes of insulin resistance, increased capacities for cell migration and invasion, and genetic alterations to understand and prevent the development of carcinogenesis induced by viral pathogenesis.

## 3. HCV Pathogenesis

The HCV positive-sense RNA genome is approximately 9.6 kb in length flanked by a 5′UTR, which includes an internal ribosome entry site (IRES), and a 3′UTR, with both participating in viral replication. The genome has a single open reading frame (ORF) that encodes a polyprotein to generate structural proteins (core, E1, and E2) and nonstructural proteins (p7, NS2, NS3, NS4A, NS4B, NS5A, and NS5B). The low-density lipoprotein receptors (LDLr) and heparin sulfate proteoglycans (HSPGs) of target cells are reported to bind HCV proteins. This triggers CD81 and the scavenger receptor B1 (SRB1) interaction with the E1/E2 heterodimer, and the virus can then enter into the cell by endocytosis [6]. The fusion of the viral and the host membranes permits the HCV RNA release into the cytosol for translation and replication. Once the viral proteins are generated, the negative-sense RNA intermediate is catalyzed by the NS5B RNA-dependent RNA polymerase and the replicated copies for the viral progeny. Finally, the newly matured virions are released into the lipoviral particles to extracellular compartments [6].

An acute HCV infection generally only causes symptoms, including weakness, anorexia, and jaundice, in 20-30% of adults. However, 70–80% of infected persons develop a chronic infection with prolonged liver damage that may cause continuous hepatocellular regeneration resulting in progressive fibrosis. If a chronic lesion persists, it can cause severe damage, leading to cirrhosis. The chronic cell turnover and progressive regeneration continue to occur and can promote hepatocarcinogenesis development [7,8]. However, to establish the direct participation of the virus and the development of cancer at the molecular level, it is important to understand each underlying molecular pathway involved in the processes of carcinogenesis and how they are regulated by the virus, to find control points as possible therapeutic targets.

## 4. Molecular Mechanisms in HCV Infection-Induced Carcinogenesis

Considering that patients with hepatitis C develop HCC after 30–40 years, that uninfected hepatocytes can also become tumor cells, and that the integration of the viral genome into the host is absent, HCV is considered oncogenic but with an indirect role in the development of carcinogenesis. However, there is a direct relationship between chronic HCV infection and the development of HCC. This suggests that the virus also plays a direct role in promoting the development of carcinogenesis, but that this is through the action of its viral proteins and the generation of a tumorigenic microenvironment into the infected cells.

**Figure 1:**
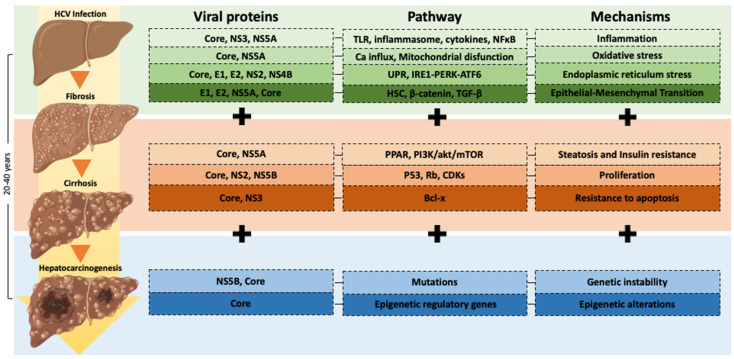
Molecular mechanisms implicated in hepatocarcinogenesis progression induced by HCV infection. The progression of HCV-infection-associated hepatocarcinogenesis involves diverse molecular mechanisms such as chronic inflammation, oxidative stress, endoplasmic reticulum stress, epithelial-mesenchymal transition, steatosis and insulin resistance, proliferation and apoptosis inhibition, genetic instability, and epigenetic alterations. Each mechanism has a specific molecular pathway regulated by viral HCV proteins.

Some mechanisms of an HCV-infection-associated hepatocarcinogenesis progression are chronic inflammation, oxidative stress, insulin resistance, steatosis, epigenetic alterations and genetic instability, epithelial-mesenchymal transition, proliferation, and apoptosis, among others. However, each mechanism has a specific molecular pathway regulated by viral HCV proteins [9] (Figure 1) (Table 2).

### 4.1. Chronic Inflammation and Oxidative Stress

**Figure 2:**
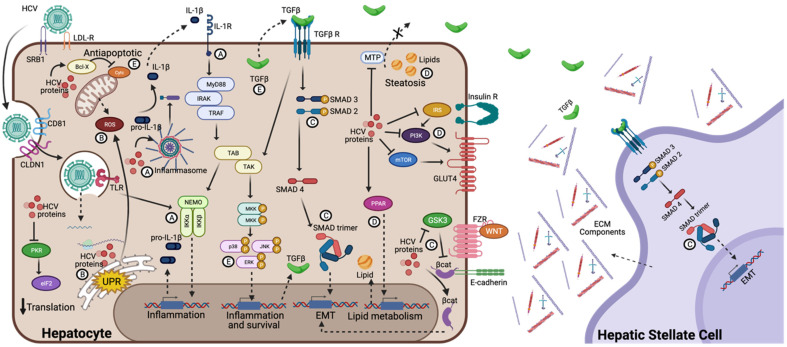
Molecular signaling pathways involved in HCV-induced hepatocarcinogenesis development. The binding and endocytosis of the HCV virus to the hepatocyte is mediated by the LDLr, CD81, and the SRB1 interaction with E1/E2 heterodimer. Once in the endosome, the HCV RNA release into the cytosol for translation and replication. The translation generates the polyprotein, and once the viral proteins are generated they can trigger diverse molecular signaling pathways, such as inflammation, by TLR and inflammasome activation (**A**); oxidative and ER stress, induced by ROS and UPR, respectively (**B**); EMT, through activation of TGF-β pathway on hepatocytes and hepatic stellate cells, and regulation of Wnt/β-catenin pathway (**C**); steatosis and insulin resistance, by inhibition of MTP and insulin receptor pathways (**D**); and proliferation and apoptosis resistance by survival expression genes and antiapoptotic proteins induction (**E**). All these simultaneous pathways induced by HCV proteins are involved in hepatocarcinogenesis development.

Once the HCV replication cycle begins in the cell, some viral proteins and replication intermediate nucleic acids are recognized in the cell membrane and cytoplasm by pattern recognition receptors (PRRs) as pathogen-associated molecular patterns (PAMPs). The activation of the PRRs induces the production of cytokines and chemokines, such as Interferon (IFN)λ1, Interleukin (IL)-1β, and the Macrophage inflammatory protein (MIP)-1, that activate the innate and adaptative immune systems [10,11,12] (Figure 2A). On the other hand, oxidative stress and reactive oxygen species (ROS) production can induce a proinflammatory gene expression, contributing to carcinogenesis. The NS5A HCV protein can enhance the Ca^+2^ influx into the mitochondria and increase ROS levels leading to the translocation of the nuclear factor kappa B (NF-κB) and the signal transducer and activator of transcription (STAT-3) into the nucleus, contributing to oxidative stress [13]. Additionally, the core protein interacts with prohibitin (a mitochondrial protein) and induces the disruption of complex I (in the mitochondrial respiratory chain) formation, leading to increases in ROS and oxidative stress [14] (Figure 2B). The activation of the immune systems are essential in fighting an HCV infection, and HCV-specific CD4+ and CD8+ T cells play an important role in the elimination of an acute HCV infection [15]. However, during the immune response, viral proteins can induce the overexpression of proinflammatory molecules such as cytokines, chemokines, and ROS, among others, through oxidative stress and the activation of PRRs. Since an HCV infection can persist for years, chronic inflammation develops, leading to an altered cellular microenvironment, with immune cell infiltration and the overexpression of pro-inflammatory molecules, which ultimately promotes cell morphological and molecular alterations leading to liver damage.

### 4.2. Epithelial-Mesenchymal Transition (EMT)

The epithelial cells exhibit plasticity under specific microenvironmental conditions, such as during embryonic development or during the physiological response to an injury. When these cells interact and form part of the surrounding mesenchyme, they lose some of their characteristics, including intercellular connections and polarity. They can also acquire new properties such as infiltration and migration capabilities. This phenomenon is called an Epithelial-mesenchymal transition (EMT). The EMT of hepatocytes can be induced by treatment with the pro-fibrogenic cytokine Transforming Growth Factor β-1 (TGF-β1). An HCV infection in vitro leads to the production of TGF-β1 through oxidative stress pathways [16]. Once the hepatocytes transition from being epithelial cells to being myofibroblasts during the EMT, they can produce extracellular matrix (ECM) components. Additionally, liver damage and continuous regeneration lead to a disproportionate ECM deposit during chronic HCV infection, ultimately inducing liver fibrosis [17]. TGF-β1 can induce the EMT, but β-catenin and Snail protein families, Twist proteins, and Zinc finger E-box-binding homeobox (ZEB) proteins also have a role in inducing the EMT and downregulating E-cadherin expression (a biomarker of the EMT) [18]. The NS5A HCV proteins can induce the EMT through the activation of Twist2 [19]. On the other hand, the core HCV viral protein regulates the Wnt/β-catenin pathway and interacts with the Snail protein, forming a complex that downregulates E-cadherin expression [20] (Figure 2C). Inflammatory processes that induce the production of some cytokines connect with some intermediate pathways of the EMT, further promoting an altered microenvironment.

**Table 2:** Molecular mechanisms by HCV proteins-induced carcinogenesis.

Molecular Mechanism	Viral Protein	Pathway	Ref.
Cell proliferation	Core	Enhancement of canonical Wnt/beta-catenin.	[21]
		Overexpression of TGF-β levels and implication of thrombospondin-1 in core-dependent TGF-β activation	[22]
	NS5A	Activation of PI3K, increased Akt/protein kinase B activity and provided protection against apoptosis	[23]
		Activation of the c-Myc promoter and increased c-Myc transcription	[24]
	NS5B	pRb is ubiquitinated and degraded in a proteasome-dependent manner	[25]
Epithelial-Mesenchymal Transition	Core	Increase Snail expression and induce EMT via STAT3	[26]
	NS5A	Activate Twist2 and induce upregulation of Vimentin and N-cadherin and downregulates E-cadherin expression	[19]
	NS5B	Induce upregulation of N-cadherin via Snail	[27]
Oxidative Stress	NS5A	Alters intracellular calcium levels, induces oxidative stress, and activates STAT3 and NF-κB.	[28]
	Core	Increases mitochondrial ROS production by stimulation of Ca^2+^ uniporter activity	[29]
Genetic alterations	NS3/NS4	Interacts with cellular protein that plays role in double-strand DNA breaks	[30]

Note: Transforming Growth Factor (TGF-β), Phosphoinositide 3-kinase (PI3K), serine/threonine protein kinase (AKT), Retinoblastoma protein (pRB), Epithelial-Mesenchymal Transition (EMT), signal transducer and activator of transcription 3 (STAT3) and nuclear factor kappa B (NF-κB).

### 4.3. EMT and Inflammasome Activation

The inflammasome is a multi-protein cytosolic complex that acts like PRR via Nod-like receptors (NLRs), and its activation induces the production of inflammatory cytokines. During an HCV infection, the NLRP3 inflammasome can sense HCV proteins and their genomes. Once active, the inflammasome recruits the apoptosis-associated speck-like protein containing CARD (ASC), which induces the activation of caspase-1 [31]. The activation of this caspase processes the pro-IL1β and pro-IL18 into mature proteins (Figure 2A). Additionally, the mature form of IL1β reaches its receptor and promotes the transcription and production of TGF-β. Once bound to its receptor, the TGF-β triggering the Smad pathway activates the transcription factor Smad4 for an EMT-related gene transcription [32]. Although hepatocytes are the liver’s main cell type, the Hepatic Stellate Cells (HSCs), pericytes found in the liver, are the major cell type involved in liver fibrosis. The activation of these cells is stimulated by TGF-β recognition, which induces its transformation to myofibroblasts. Similar to hepatocytes after the EMT, HSC can induce the production of ECM components, ultimately inducing liver fibrosis [33] (Figure 2C). In addition to inflammation and the EMT that lead to an altered extracellular microenvironment, multiple molecular pathways inside the cell, such as apoptosis, cell cycle, and endoplasmic reticulum stress, among others, are also altered and affect appropriate hepatocyte function.

### 4.4. Steatosis and Insulin Resistance

Hepatic steatosis is characterized by an accumulation of lipid droplets (LDs) in the cell’s cytoplasm, and steatosis is an additional risk for the development of hepatocellular carcinoma. HCV can induce steatosis by reducing the secretion of lipids and altering lipid metabolism in the infected hepatocytes. The overexpression of the core protein can reduce microsomal triglyceride transfer protein (MTP) activity and very low-density lipoprotein secretion [34]. Lipid accumulation by HCV has proposed fatty acid synthesis upregulation mechanisms by sterol regulatory element-binding protein-1 (SREBP1) activation. However, several mechanisms in HCV-induced steatosis must be elucidated. On the other hand, HCV can induce insulin resistance by altering insulin signaling by alterations on the PI3K/Akt/mTOR pathways and the degradation of receptor substrate 1 (IRS-1) [35] (Figure 2D). Furthermore, the HCV core protein induces insulin resistance by altering protein phosphorylation with the subsequent alteration of the mTOR/S6K1 pathway and glucose uptake [36]. Additionally, NS5A induces gluconeogenic gene expression (G6P, PCK2), resulting in an increased glucose production that enhances insulin resistance [37]. In addition to altering cellular functionality, HCV can induce cellular stress by exceeding the functional capacity of the organelles.

### 4.5. Endoplasmic Reticulum Stress

**Figure 3:**
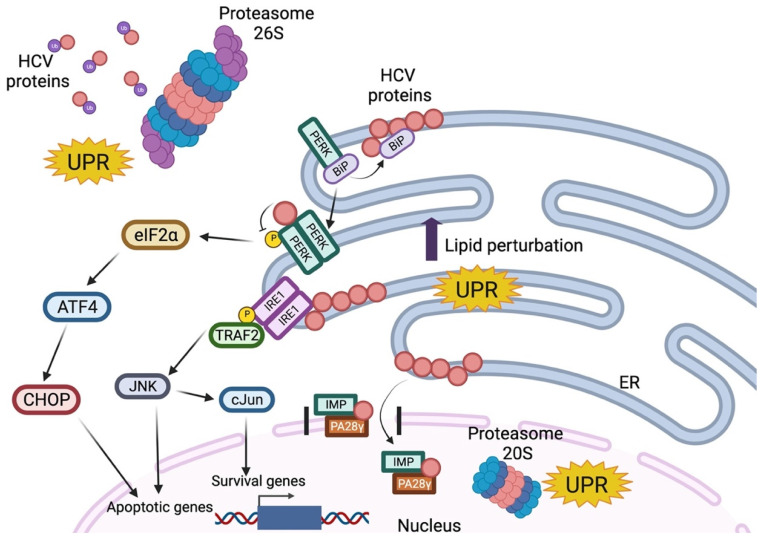
Unfolded Protein Response induced by HCV proteins. Normally BiP is attached to PERK in an inactive state. Unfolded and/or HCV proteins sequester BiP from PERK, allowing its oligomerization and auto-phosphorylation. Activated PERK (which phosphorylates eIF2α to inhibit global protein synthesis) is inhibited by viral proteins in the cytosol. This prevents the translation of ATF4, which activates CHOP, a pro-apoptotic transcription factor. On the other hand, the viral proteins can activate IRE1 and recruit TRAF2 to initiate a cascade of phosphorylation of JNK (pro-apoptotic) and c-Jun (pro-survival). Meanwhile, mature HCV proteins release to the cytoplasm where they can be polyubiquitinated (Ub) and degraded by proteasome 26S. Simultaneously, some viral proteins can be imported into the nucleus by their association with PA28 and IMP, which are degraded by proteasome 20S independent of ubiquitin. All these molecular pathways are involved in the unfolded protein response, which finally regulates some survival, inflammation, and apoptotic genes.

During an HCV infection, structural and non-structural proteins are localized inside or integrated into the endoplasmic reticulum (ER), which causes specific modifications in the ER [38]. The ER’s main function is to modify and fold newly synthesized proteins, and ER stress occurs when the number of proteins in the ER exceeds its folding capacity. The unfolded protein response (UPR) is an auto-protective response to reduce the protein load and regulate protein folding by decreasing protein synthesis while upregulating the chaperone’s expression. The molecular pathway of the UPR is initiated by IRE1, PRKR-like endoplasmic reticulum kinase (PERK), and Activating Transcription Factor (ATF)6 activation. IRE1 splices the mRNA of the transcription factor X-box-binding protein (XBP)-1; PERK activation downregulates the protein synthesis by phosphorylation of eIF2; ATF6 translocates to the Golgi apparatus cleavage to release into the cytosol; and is finally transported to the nucleus to induce the increased expression of chaperones [39] (Figure 3). The expression of the HCV replicon induces the splicing of XBP-1 mRNA, but the target genes of XBP-1 are not elevated, suggesting that HCV manipulates the UPR pathway. It was also shown that the ER stress biomarkers were elevated by expressing core, E1, E2, NS2, and NS4B HCV proteins [40]. For example, Tardif et al. demonstrated that NS4B induces ATF6 cleavage and XBP1 splicing [41]. On the other hand, core, NS2, and NS5B proteins interact with the proteasomal pathways proteins and alter the function that elicits the UPR leading to ER stress [42]. Although the UPR is related to apoptosis, chronic ER stress is also capable of inducing the altered adaptation, leading to cell survival and proliferation.

### 4.6. Proliferation and Apoptosis

Cyclin-dependent kinases (CDKs) and cyclins control the cell division in all cells by regulating the transitions of cells toward each cell cycle phase. Complexes with cyclin E, cyclin A, and CDK2 facilitate the progression of the S phase the inhibitors of these proteins participate in regulating the cell cycle, such as with Rb, E2F-1, DP-1, p107, and p130 [43]. The alteration of cell proliferation in some viral infections is induced by the viral protein’s actions on CDKs or cyclins. This alteration and antiapoptotic signals prompt the development of carcinogenesis. Some studies associated HCV proteins with proliferative signaling alterations. Core proteins modulate the mitogen-activated protein kinase, promoting hepatocyte proliferation and downregulating CDKN2A to overcome senescence [44]. Furthermore, the HCV core protein module p21/Waf (a putative tumor suppressor) expression binds to p53 and Rb, which are involved in cell cycle control [45]. Additionally, NS5B induces Rb degradation by ubiquitination, activates E2F-responsive promoters, and induces cell proliferation [46]. In addition, NS5B delays S-phase progression by interacting with the cyclin-dependent kinase 2-interacting protein (CINP) [47]. On the other hand, HCV proteins also modulate apoptosis signaling pathways. The core protein induces the activation of Bcl-X and prevents the release of cytochrome C from mitochondrial and consecutive caspases activation [48,49]. Additionally, NS3 prevents viral RNA-induced pro-apoptotic RIG-I effects [50] (Figure 2E). HCV is able to control cell death and survival, and it is crucial for the development of altered cells that, in addition to being highly proliferative, tend to present genetic instability.

### 4.7. Epigenetic and Genetic Alterations

Hepatocellular carcinoma can progress through the deregulation of complex epigenetic networks [51]. Alterations in gene methylation are related to several virus-induced cancers. The inactivation of tumor suppressor genes by hypermethylation, as well as the activation of oncogenes by demethylation generated by an HCV infection, greatly promotes the development of carcinogenesis. In hepatocellular carcinoma, tumor suppressor genes such as Glutathione S-transferase P (GSTP1), Runt-related transcription factor (RUNX)3, APC, and CDKN2 are highly methylated by both HBV and HCV infections [52]. Pin Zhao et al. reported alterations in the methylation status of 18 specific genes that have been associated with different molecular pathways, including the Wnt signaling pathway, the Ras/MAPK signaling pathway, the p53 signaling pathway, the JAK-STAT pathway, and the PI3K-AKT induced by an HCV infection (Table 3) [51]. Furthermore, the aberrant regulation of histone modifications is also related to hepatocellular carcinogenesis associated with HCV infections, especially histone acetylation for oncogene activation and methylation for tumor suppressor gene silencing.

On the other hand, two common repetitive elements associated with cancer in humans, the long interspersed nuclear element-1 (LINE-1) and the Alu element (Alu), have a loss of methylation in hepatocellular carcinoma induced by HCV [63]. Additionally, p16, Ras association domain family 1 isoform A (RASSF1A), GSTP1, and Retinoblastoma protein-interacting zinc-finger gene 1 (RIZ1) are genes with aberrant methylation levels in HCV infections associated with hepatocellular carcinoma [64]. The accumulation of aberrant DNA methylation and hypomethylation on tumor suppressor genes predisposed to cancer development.

However, the direct action of HCV viral proteins on these epigenetic mechanisms remains unclear. Recent studies have focused on miRNAs and LncRNAs for searching for genetic alterations. For example, miR-122 has been reported to be involved in HCV replication and is associated with HCC development. Additionally, miR-122-5p, miR-222-3p, miR-146-5p, miR-150-5p, miR-30C-5p, miR-378a-3p, and miR-20a-5p have been shown to be enriched in exosomes derived from HCV-infected cells. Furthermore, it has been revealed that the levels of these liver-specific miRNAs showed a significant decrease after DAA therapy. Additionally, miRNA-122 has been considered as an early biomarker for HCC development [65].

Taken together, the molecular mechanisms of HCV-induced carcinogenesis through a complex epigenetic network also play an important role for supporting the development of effective antiviral treatments that help patients respond to infection; however, in many cases, carcinogenesis continues to occur even when the viral infection has been resolved.

## 5. Carcinogenesis after Clearance of HCV Infection

Interferon-alpha (IFN-α) and its analog, pegylated IFN-α (PEG-IFN-α), alone or in combination with ribavirin (RBV), were the main antiviral treatments for HCV infections for many years. However, the therapeutic regimens based on the use of IFN were characterized by a sustained virologic response (SVR) of 40–50%. Continuous HCV studies have developed new drugs called direct-acting antivirals (DAAs) which are characterized by a sustained virologic response (SVR) of 98% [65]. Nevertheless, patients treated with new DAAs have reported HCC development in 3–7% of cases, even with SVR [66,67]. These data have initiated a high debate about the risk of developing HCC after the clearance of an HCV infection by DAAs, and even the potential role of DAAs in carcinogenesis [68].

Several hypotheses have been made to explain this: the presence of cancer nodules before the treatment, the induction of carcinogenesis by the DAAs, or epigenetic alterations post-clearance induced by an HCV infection. As we know, carcinogenesis is induced by several indirect and direct mechanisms of an HCV infection, and the clearance of infection does not lead to the reversal state of advanced liver damage, which by itself is a potential risk factor for developing HCC. One possible direct role of DAAs is in reducing immunosurveillance in response to the rapid decrease in viral load. Antiproliferative IFN-stimulated genes showed a downregulation after infection clearance by DAAs. Additionally, a higher VEGF expression and alteration of inflammatory and anti-inflammatory cytokines balance was shown after treatment with DAAs [69,70]. On the other hand, it is thought that HCV may exert a direct action on the risk of developing HCC after virus clearance.

As seen, HCV proteins have several direct ways to induce carcinogenesis, such as through epigenetics and genetic alterations. Nevertheless, the study of these alterations after an HCV infection is still unclear. One study demonstrated that HCV modifies histone modification positions and induces epigenetic alterations related to the development of HCC that persist with the clearance of the infection using DAAs [71]. In addition, patients treated with IFN or DAAs had similar results and showed changes in histone H3K27ac that were still present after HCV clearance [72]. However, the molecular mechanisms of HCC development after the clearance of an HCV infection are presently unknown, with no supporting evidence of the DAAs’ direct role. New insights in research are required to explain the molecular mechanisms by which HCV induces carcinogenesis even after SVR with DAAs.

## 6. Conclusions

Knowledge of the molecular mechanisms induced by viruses make it possible that effective vaccines can be developed in a relatively short period of time to prevent some diseases. However, decades of HCV studies on the molecular mechanisms of pathogenesis have not been sufficient to completely understand how an HCV infection can induce carcinogenesis. An HCV infection remains one of the main causes of the development of HCC. Despite the use of specific antivirals, patients treated with new DAAs reported an unexpected increase in the early incidence of HCC. Some direct and indirect mechanisms of the progression of hepatocarcinogenesis associated with an HCV infection are chronic inflammation (TLR and inflammasome activation), oxidative and ER stress (ROS and UPR, respectively), an EMT (TGF-β pathway on hepatocytes and hepatic stellate cells), steatosis and insulin resistance (the inhibition of MTP and insulin receptor pathways) and proliferation and apoptosis resistance (the survival expression of genes and the induction of antiapoptotic proteins). The orchestrated regulation of these cellular pathways induced by HCV proteins is involved in the development of hepatocarcinogenesis. Nevertheless, it is necessary to extend the panorama and better understand the development of carcinogenesis before, during, and after resolution of a viral infection. Then, we can identify cellular and molecular checkpoints to be used as potential therapeutic targets.

## Figures and Tables

**Table 1:** Human oncoviruses-associated cancer cases.

Virus	Characteristics	Oncogenic Findings	Attributable Cancer Cases
EBV	Enveloped and linear DNA. Tropism: Epithelium and B cell.	Oncoprotein LMP1 induces proliferation and resistance to apoptosis. Encodes virally microRNAs.	55% of Burkitt’s lymphoma, 50% of Hodgkin’s lymphoma and 84% of Nasopharyngeal carcinoma [1].
HBV	Enveloped and circular partial DNA. Tropism: Hepatocytes.	Chronic inflammation, Tissue injury, oncoprotein HxB induces oxidative DNA damage, metastasis, and proliferation pathways. Insertion of viral genome into host DNA.	55% Hepatocellular carcinoma [1].
HCV	Enveloped and positive-sense RNA. Tropism: Hepatocytes.	Chronic inflammation, tissue injury, NS5A induce ER stress, Core protein induce steatosis and insulin resistance.	21% of Hepatocellular carcinoma and 3% Non-Hodgkin lymphomas [1].
HPV	Naked and circular DNA. Tropism: Stratified squamous epithelium	Viral genome insertion into host DNA, Oncoproteins E6 and E7 manipulate cell cycle and inhibit apoptosis, E5 induce proliferation pathways.	100% of Cervix, 30% of Oropharynx, 53% of Penile, 77% of Vaginal and 25%of Vulvar [1].
HTLV-1	Enveloped and positive-sense RNA. Tropism: T and B cells	Oncoprotein Tax promotes viral replication and activate proliferation, senescence, and genomic instability pathways.	100% of T-cell leukemia and Lymphoma [1].
KSHV	Enveloped and linear DNA. Tropism: Oropharyngeal epithelium	Encodes viral interleukins and chemokines which promotes proliferation and angiogenesis. Oncoprotein K1 induce cell transformation. LANA protein inhibits cell cycle checkpoints.	100% of Kaposi’s sarcoma [1]
MCPyV	Naked and circular DNA. Tropism: Skin	T-antigen induce cell proliferation and cell transformation. Insertion of viral genome into host DNA	80% of Merkel cell carcinoma [5].

Note: Epstein–Barr virus (EBV), hepatitis B virus (HBV), Hepatitis C virus, human papillomaviruses (HPV), human T-cell lymphotropic virus (HTLV-1), Kaposi’s sarcoma-associated herpesvirus (KSHV), and Merkel cell polyomavirus (MCPyV).

**Table 3:** Epigenetic modifications associated with hepatocarcinogenesis induced by HCV infection.

Methylation Status	Type Genes	Genes	Molecular Mechanisms involved	Ref.
Hypermethylated	Tumor suppressor	RASL1, EGLN3, CSMD1, CDKN2A, BCORL1, SFRP1, P73, ZNF382, RUNX3, LOX, RB1	Proliferation, tumor growth inhibition, cell cycle regulation, apoptosis, EMT.	[51,53,54,55,56,57,58]
Hypomethylated	Oncogenic	OTX2, IGF1R, SNCG, ZBTB16, FOXA1, HNF4A, CEBPA	Increasing fibrosis, preneoplastic alterations, EMT, apoptosis inhibition, lipid metabolism, proliferation, inflammation, cell motility.	[51,59,60,61,62]

Note: RAS protein activator like 1 (RASAL1), Egl-9 family hypoxia inducible factor 3 (EGLN3), CUB and Sushi multiple domains 1 (CSMD1), cyclin-dependent kinase inhibitor 2A (CDKN2A), BCL6 corepressor like 1 (BCORL1), Secreted frizzled-related protein 1 (SFRP1), Zinc-finger protein 382 (ZNF382), RUNX family transcription factor 3 (RUNX3), Lysyl oxidase (LOX), orthodenticle homeobox 2 (OTX2), Type 1 receptor of insulin-like growth factor (IGF1R), neuronal protein, synuclein-gamma (SNCG), Zinc Finger and BTB Domain Containing 16 (ZBTB16), Forkhead boxA1 (FOXA1), Hepatocyte nuclear factor 4a (HNF4a), CCAAT/enhancer-binding protein alpha (CEBPA).

## Data Availability

Not applicable.
